# Effect of *attC *structure on cassette excision by integron integrases

**DOI:** 10.1186/1759-8753-2-3

**Published:** 2011-02-18

**Authors:** André Larouche, Paul H Roy

**Affiliations:** 1 Centre de Recherche en Infectiologie, Centre Hospitalier Universitaire de Québec, Québec, Canada; 2 Département de Biochimie, de Microbiologie et de Bio-informatique, Faculté des Sciences et de Génie, Université Laval, Québec, Canada

## Abstract

**Background:**

Integrons are genetic elements able to integrate and disseminate genes as cassettes by a site-specific recombination mechanism. These elements contain a gene coding for an integrase that carries out recombination by interacting with two different target sites; the *attI *site in *cis *with the integrase and the palindromic *attC *site of a gene cassette. Integron integrases (IntIs) bind specifically to the bottom strand of *attC *sites. The extrahelical bases resulting from folding of *attC *bottom strands are important for the recognition by integrases. These enzymes are directly involved in the accumulation and formation of new cassette arrangements in the variable region of integrons. Thus, it is important to better understand interactions between IntIs and their substrates.

**Results:**

We compared the ability of five IntIs to carry out excision of several cassettes flanked by different *attC *sites. The results showed that for most cassettes, IntI1 was the most active integrase. However, IntI2*179E and SonIntIA could easily excise cassettes containing the *attC_dfrA1 _*site located upstream, whereas IntI1 and IntI3 had only a weak excision activity for these cassettes. Analysis of the secondary structure adopted by the bottom strand of *attC_dfrA1 _*has shown that the identity of the extrahelical bases and the distance between them (A-N_7-8_-C) differ from those of *attC*s contained in the cassettes most easily excisable by IntI1 (T-N_6_-G). We used the *attC_dfrA1 _*site upstream of the *sat2 *gene cassette as a template and varied the identity and spacing between the extrahelical bases in order to determine how these modifications influence the ability of IntI1, IntI2*179E, IntI3 and SonIntIA to excise cassettes. Our results show that IntI1 is more efficient in cassette excision using T-N_6_-G or T-N_6_-C *attC*s while IntI3 recognizes only a limited range of *attC*s. IntI2*179E and SonIntIA are more tolerant of changes to the identity and spacing of extrahelical bases.

**Conclusions:**

This study provides new insights into the factors that influence the efficiency of cassette excision by integron integrases. It also suggests that IntI2 and SonIntIA have an evolutionary path that is different from IntI1 and IntI3, in their ability to recognize and excise cassettes.

## Background

In recent years, Gram-negative pathogens such as *Pseudomonas aeruginosa*, *Acinetobacter baumannii*, and *Klebsiella pneumoniae *have become increasingly resistant to antibiotics. The widespread dissemination of bacterial resistance genes is mediated by horizontal transfer and many of these genes are integrated and expressed as operons in DNA elements called integrons.

**Figure 1:**
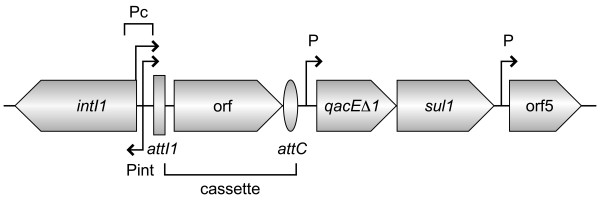
**General structure of class 1 integrons**. Cassettes are inserted in the variable region of integrons by a site-specific recombination mechanism. The *attI1 *and *attC *sites are shown by a vertical rectangle and oval, respectively, and promoters are denoted by Pint, Pc and P. Integrated cassettes are composed of a gene and an *attC *recombination site. Genes are as follows: *intI1*, integrase gene; *qacEΔ1*, antiseptic resistance gene; *sul1*, sulphonamide resistance gene; orf5, gene of unknown function.

Integrons are genetic elements that can integrate and disseminate genes as cassettes by a site-specific recombination mechanism [1]. They contain an integrase gene (*intI*), a recombination site (*attI*), and a promoter region (P_c_) that directs the expression of captured genes (Figure 1) [2]. Cassettes located within the variable region of integrons all share certain characteristics. First, the integrated cassettes are composed of a gene and an imperfect inverted repeat, called an *attC *site, located downstream of the gene (Figure 1) [3-6]. Second, the boundaries of each integrated cassette are defined by two GTTRRRY sequences that are targets for recombination events mediated by integron integrases (IntIs).

Studies on the site-specific recombination mechanism mediated by IntIs have demonstrated that IntIs form a separate subfamily, characterized by the presence of an additional domain required for their activity, within the larger family of tyrosine recombinases [7,8]. IntIs can share as little as 35% sequence identity, indicating a long evolutionary history for these enzymes. Their catalytic domain is similar to that of other members of the tyrosine recombinase family and contains the conserved residues: Arg_146_-Lys_171_-His_277_-Arg_280_-His/Trp_303 _and the nucleophilic tyrosine, Tyr_312 _(coordinates are those of IntI1).

Unlike other members of the family, the IntI recombinases can exchange DNA using two sites with different structures, the non-palindromic *attI *and palindromic *attC*. Integration of cassettes occurs preferentially by recombination of the *attC *site in a closed-circular cassette with the *attI *site of an integron [3] while excision of a cassette, generating a circular form, occurs preferentially by recombination between two *attC *sites, one of them associated with the upstream cassette [4].

**Figure 2:**
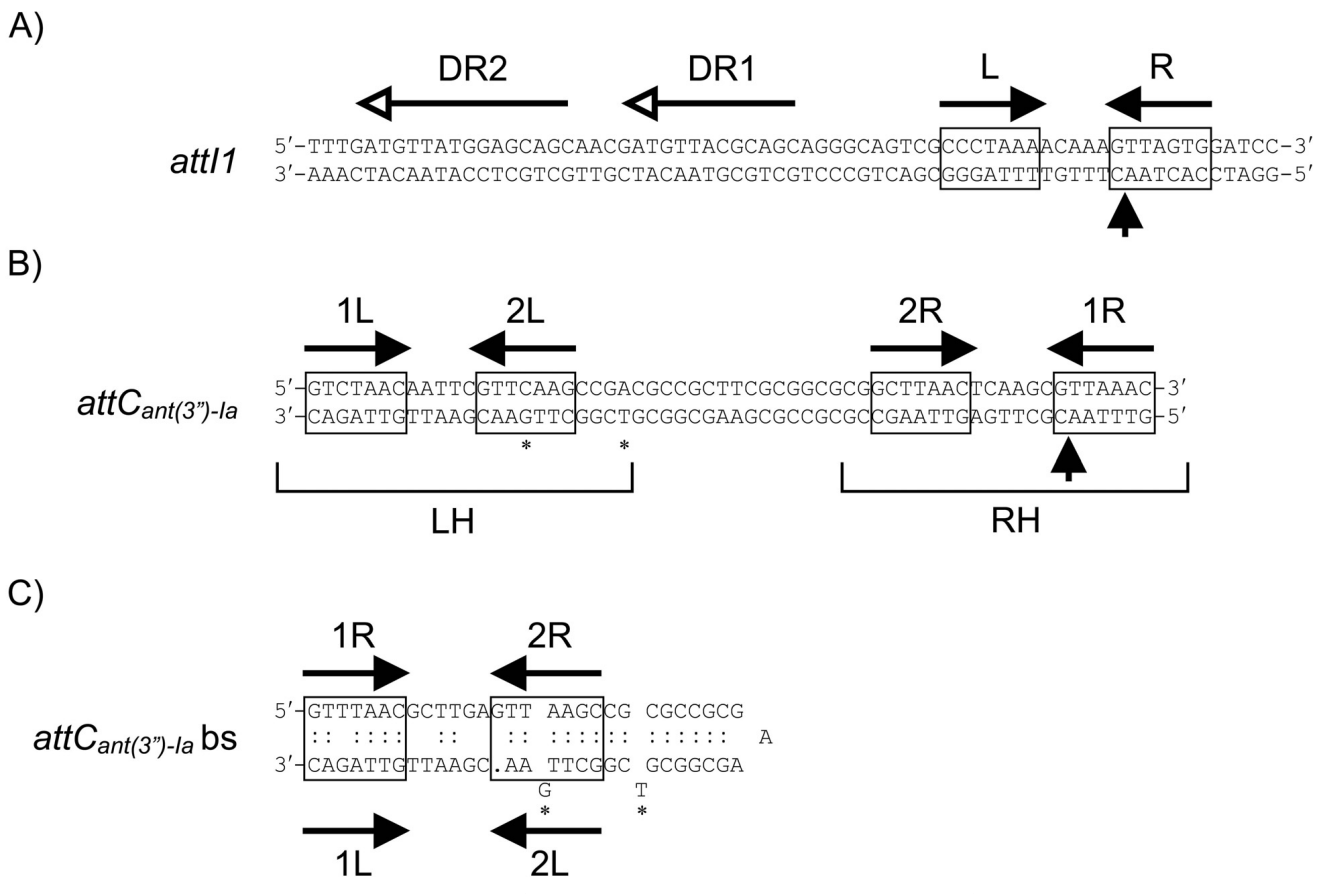
**Integron recombination sites**. (a) Sequence of the double strand (ds) *attI1 *site. (b) Sequence of the ds *attC_ant(3'')-Ia _*site. (c) Secondary structure of the folded bottom strand of the *attC_ant(3'')-Ia _*site, according to MFOLD (http://mfold.bioinfo.rpi.edu/cgi-bin/dna-form1.cgi). The inverted repeats L, 1L, and 2L, R, 1R and 2R are shown by horizontal black arrows. The *attI1 *direct repeats bound by IntI1 are indicated by horizontal lines with an empty arrowhead. The crossover positions are indicated by vertical arrows and the extrahelical bases are identified by asterisks.

The *attI *and *attC *sequences are complex attachment sites that include the crossover site and additional binding sites (Figure 2), suggesting that integrase monomers act as accessory factors at these additional sites [9-11]. *attI *sites are located at the end of the 5' conserved region of integrons and their sequences vary considerably. Unlike the *attI *sites, *attC *sites share a common set of characteristics that enable them to be identified despite the diversity of their sequence and size [6,12]. They are characterized by a palindrome of variable length and sequence between the RYYYAAC inverse core site and the GTTRRRY core site [12]. The size of these recombination sites (57 to 141 bp) is currently the main criterion for classification of *attCs *[13,14]. They consist of two pairs of binding sites in opposite orientation (1L-2L and 2R-1R), each pair forming a simple site (LH and RH), separated by a segment of variable length and sequence but including an inverted repeat (Figure 2) [12]. These features are generally well recognized by IntI enzymes since many *attC *sites can act as recombination sites for IntIs sharing less than 50% amino acid sequence identity [6,12,15-20].

**Figure 3:**
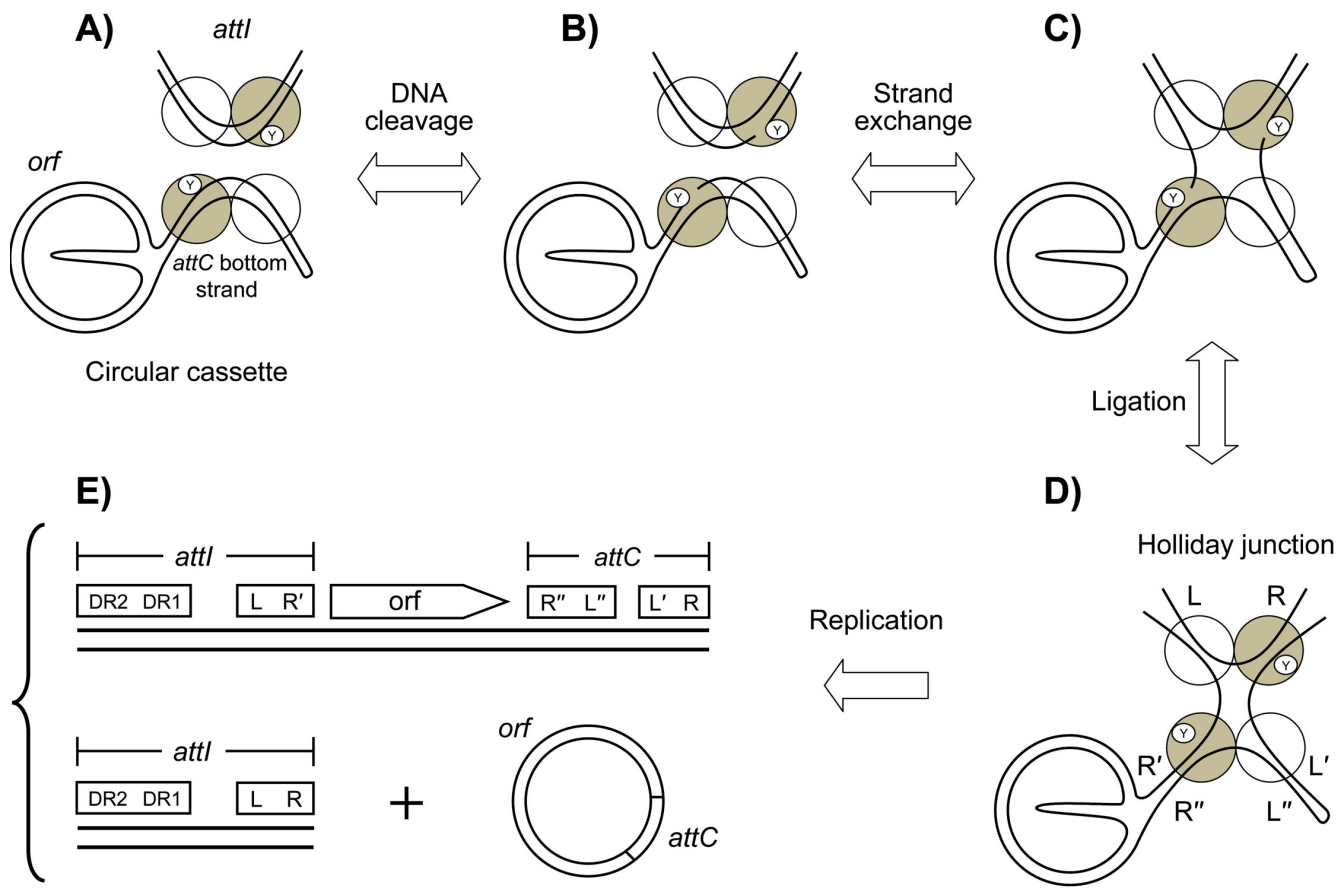
**Model of cassette integration mediated by integron integrases**. The site-specific recombination reaction is carried out between the folded bottom strand of an *attC *site and the *attI *site of an integron. (a) Two integrase monomers bind each DNA molecule. (b) Attacking monomer on each DNA molecule cuts one of the two strands. (c) Strand exchange of cut strands. (d) Ligation of exchanged strands forms a Holliday junction. (e) This intermediate structure could be resolved by DNA replication, generating three products, one of which contains the inserted cassette. The attacking monomers are shown by gray circles and the non-attacking monomers are shown by white circles. Y: catalytic tyrosine residues. Adapted from [28].

As members of the tyrosine recombinase family, IntIs use a topoisomerase I type mechanism of cleavage [8,21,22]. Four integrase monomers are involved in the site-specific recombination reaction in which the exchange of one DNA strand contributes to the formation of a Holliday junction [23,24]. For most tyrosine recombinases, this intermediate is resolved by the exchange of the second strand [22,25]. IntIs differ from other tyrosine recombinases by their use of a folded single-stranded *attC *site [26,27] and by the exchange of one strand, with the intermediate possibly resolved by DNA replication (Figure 3) [28,29].

**Figure 4:**
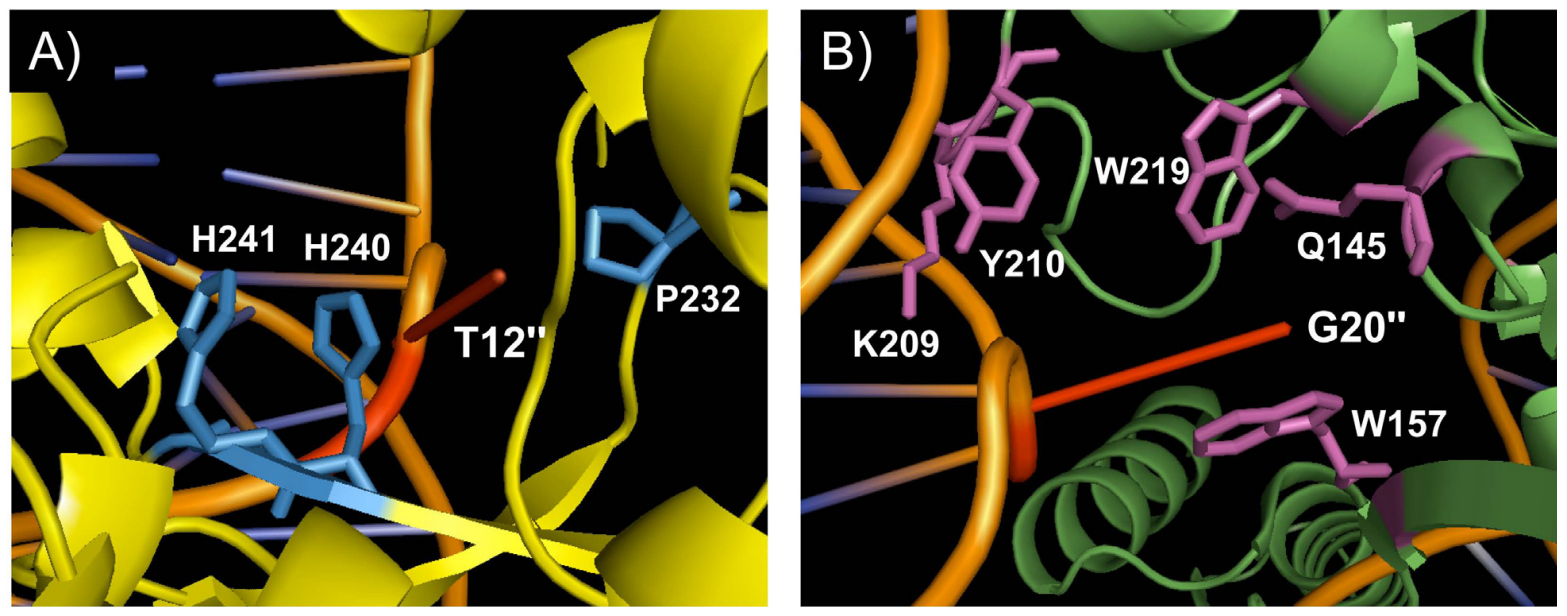
***cis *and *trans *extrahelical base interactions**. (a) *cis *interactions of the VchIntIA non-attacking subunit made by P232, H240 and H241 (in blue) with the extrahelical base T12''. (b) *trans *interactions of the VchIntIA attacking subunit made by Q145, W157, K209, Y210 and W219 (in magenta) with the extrahelical base G20''. The non-attacking subunit is in yellow, the attacking subunit in green, DNA in orange, and the extrahelical bases T12'' and G20'' are in red. (Based on the structure of the VchIntIA-VCR_bs _complex (PDB:2A3V) [29].

IntI recombinases bind specifically to the bottom strand (bs) of *attC *[26] and the extrahelical bases resulting from folding of the *attC *bs are important for recognition by IntIs [27,29,30]. The VchIntIA-*Vibrio cholerae *repeat (VCR)_bs _three-dimensional structure showed that the β-4,5 strands from the non-attacking subunits interact with the extrahelical base T12" (first extrahelical base of the folded *attC *bs) while the α-I_2 _helix from the attacking subunits forms several important contacts in *trans *with DNA in the region of the extrahelical base G20" (second extrahelical base of the folded *attC *bs; Figure 4A and 4B) [29].

To date, more than 100 IntIs have been reported in the literature and databases and it is estimated that about 10% of partially or completely sequenced bacterial genomes carry genes coding for these enzymes [31]. Activity for cassette excision and integration has been demonstrated for IntI1 [5,16,32], IntI2*179E [18], IntI3 [15], SonIntIA [17], NeuIntIA [19] and VchIntIA [32]. Moreover, it has been shown that IntIs can excise cassettes containing a variety of *attC *sites [17,19,33]. However, it is not well understood why these enzymes can easily recognize and excise some cassettes, while others are poorly (or not) excised.

We compared the ability of several IntIs to excise cassettes flanked by different *attC *sites. Preliminary results of these excision tests combined with molecular modelling based on the structure of the VchIntIA integrase leads us to suggest that IntIs prefer certain *attC *sites to others and that these preferences could be related to the recognition of the extrahelical bases. In this study, we used the *attC_dfrA1 _*site upstream of the *sat2 *cassette as a template to alter nucleotide sequence and spacing between the extrahelical bases in order to determine how these modifications influence the efficiency of cassette excision by IntI1, IntI2*179E, IntI3 and SonIntIA.

## Results

### Comparative excision activities of IntI1, IntI3, IntI2*179E, SonIntIA and VchIntIA on cassettes containing different *attC *sites

**Figure 5:**
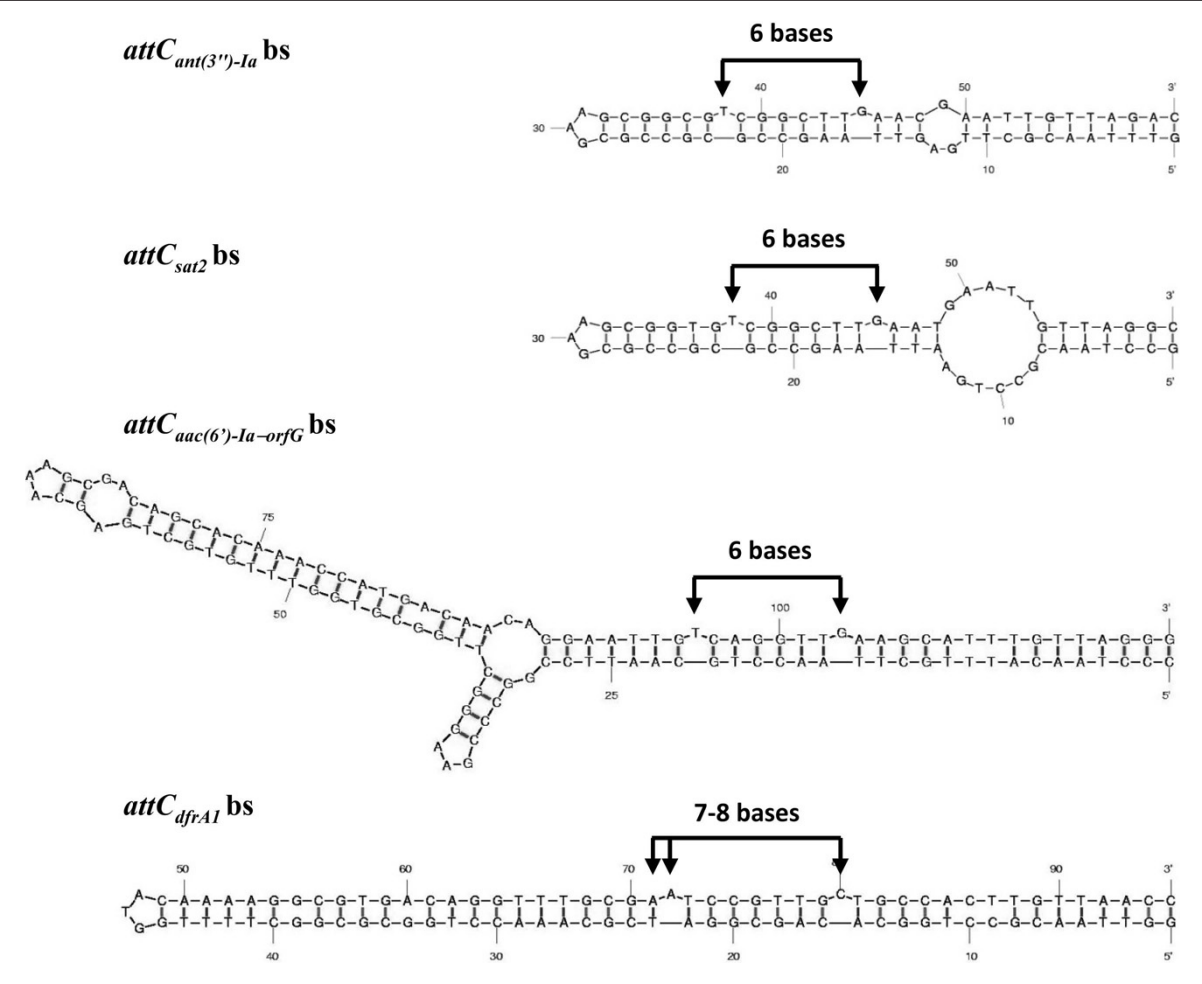
**Secondary structure of the folded bottom strand of the *attC_ant(3'')-Ia_*, *attC_sat2_*, *attC_aac(6')-Ia-_*_orfG _and *attC_dfrA1_*sites**. The extrahelical bases are identified by arrows.

In order to determine why some cassettes are excised by several IntIs while others are poorly (or not) excised, we compared the efficiency of five IntIs in excision of cassettes flanked by different *attI *and *attC *sites. Nineteen clones (pLQ423 to pLQ431 and pLQ437 to pLQ446 (Table 1) containing various resistance gene cassettes cloned into pACYC184 were used to compare the recombination activity of IntI1, IntI3, IntI2*179E, SonIntIA and VchIntIA by qualitative excision tests (QL-ETs). The results showed a pronounced effect of the identity and spacing of the extrahelical bases in the *attC *sites on the efficiency of cassette excision. All integrases efficiently excised cassettes flanked by *attC *sites whose extrahelical bases are T and G separated by a distance of six nucleotides, with some exceptions for VchIntIA. IntI1 and IntI2*179E also efficiently excised cassettes with their homologous *attI *site upstream and this same *attC *site downstream. IntI1 was also able to recognize *attI2 *but IntI2*179E was unable to recognize *attI1*. Notably, IntI2*179E and SonIntIA could easily recognize and excise cassettes with the *attC_dfrA1 _*site located upstream of the cassette, whereas IntI1 and IntI3 had only a weak excision activity for the same cassettes and the VchIntIA integrase did not excise any of them. The *attC_dfrA1 _*folded bs has the extrahelical bases A71 or A72 (either of these adenines could pair with the thymine at position 23) and C80 separated by a distance of 7 or 8 nucleotides (Figure 5). The unusual specificity shown by IntI2*179E and SonIntIA led us to choose clone pLQ430, with *attC_dfrA1 _*upstream of the *sat2 *cassette (with its T-N_6_-G-containing *attC *site downstream), for tests of the effect of changes of the upstream *attC_dfrA1 _*site on efficiency of cassette excision by the various integrases.

**Table 1:** Results obtained from the qualitative excision tests

Clone pLQ	Upstream *att *site	Cassette	Integron integrases (IntIs)				
			
			IntI1	IntI3	IntI2*179E	SonIntIA	VchIntIA
423	*attI1*	*ant(3'')-Ia *T-N_6_-G	++	-	-	-	-

424	*attI2*	*dfrA1 *A-N_7-8_-C	-	-	+	-	-

425	*attI3*	*bla_IMP-1 _*T-N_6_-G*	-	-	-	-	-

426	*attC_dfrA1 _*A-N_7-8_-C	*ant(3'')-Ia *T-N_6_-G	+	-	+	+	-

427	*attI1 *N/A	*dfrA1 *A-N_7-8_-C	-	-	-	-	-

428	*attC_ant(3'')-Ic _*T-N_6_-G	*aac(6')-Ia*-orfG + orfH T-N_6_-G	+++	+++	+++	+++	+++

429	*attI1 *N/A	*ant(3'')-Ic *T-N_6_-G	++	-	-	-	-

430	*attC_dfrA1 _*A-N_7-8_-C	*sat2 *T-N_6_-G	+	+	++	++	-

431	*attC_aac(6')-Ib _*TC-N_6_-G	*bla_oxa10 _*T-N_6_-G	-	-	-	-	-

437	*attC_ant(3'')-Ic _*T-N_6_-G	*dfrA1 *A-N_7-8_-C	-	-	+	-	-

438	*attC_ant(3'')-Ic _*T-N_6_-G	*bla_IMP-1 _*T-N_6_-G*	+++	+++	+++	+++	-

439	*attI1 *N/A	*bla_IMP-1 _*T-N_6_-G*	+	-	-	-	-

440	*attI1 *N/A	*aac(6')-Ia*-orfG + orfH T-N_6_-G	+++	-	-	-	-

441	*attI2 *N/A	*aac(6')-Ia*-orfG + orfH T-N_6_-G	++	-	+++	+	-

442	*attI3 *N/A	*aac(6')-Ia*-orfG + orfH T-N_6_-G	+	-	-	-	+

443	*attC_ant(3'')-Ic _*T-N_6_-G	*ant(3'')-Ia *T-N_6_-G	+++	+++	+++	+++	+++

444	*attC_dfrA1 _*A-N_7-8_-C	*dfrA1 *A-N_7-8_-C	+	+	+++	+++	-

445	*attC_aac(6')-Ia_*_-orfG _T-N_6_-G	*ant(3'')-Ia *T-N_6_-G	+++	+++	++	++	++

446	*attC_blaIMP-1 _*T-N_6_-G	*aac(6')-Ia*-orfG + orfH T-N_6_-G	+++	+++	+++	+++	-

-, No excision; +, weak excision (<20%); ++, moderate excision (20 to 75%); +++, strong excision (>75%); *, additional secondary structure between the extrahelical bases.

### Comparative excision activities of IntI1, IntI3, IntI2*179E and SonIntIA on cassettes with an upstream *attC_dfrA1 _*site or mutant *attC_dfrA1 _*sites

We then determined the effect of different *attC *structures on excision by IntI1, IntI3, IntI2*179E and SonIntIA. We made several mutants of the *attC_dfrA1 _*site, upstream of the *sat2 *cassette in pLQ430, with altered extrahelical base identity and spacing, and used QN-ETs.

**Figure 6:**
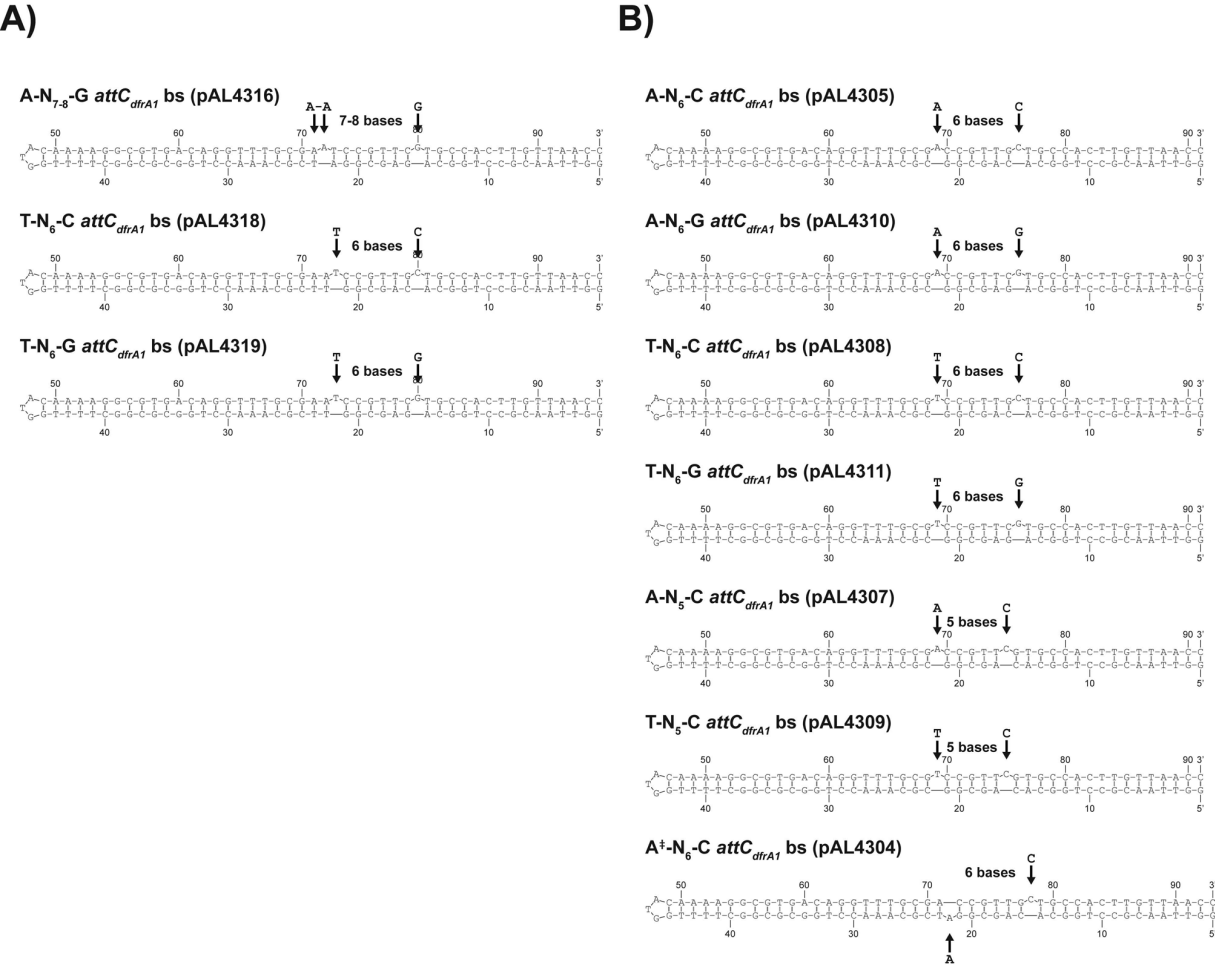
**Secondary structure of the folded bottom strand of several mutants of the *attC***_***dfrA1***_**site**. (a) First set of mutants. (b) Second set of mutants. The extrahelical bases are identified by arrows. The ‡ indicates that the first extrahelical base is located on the opposite side of the folded bottom strand structure.

**Figure 7:**
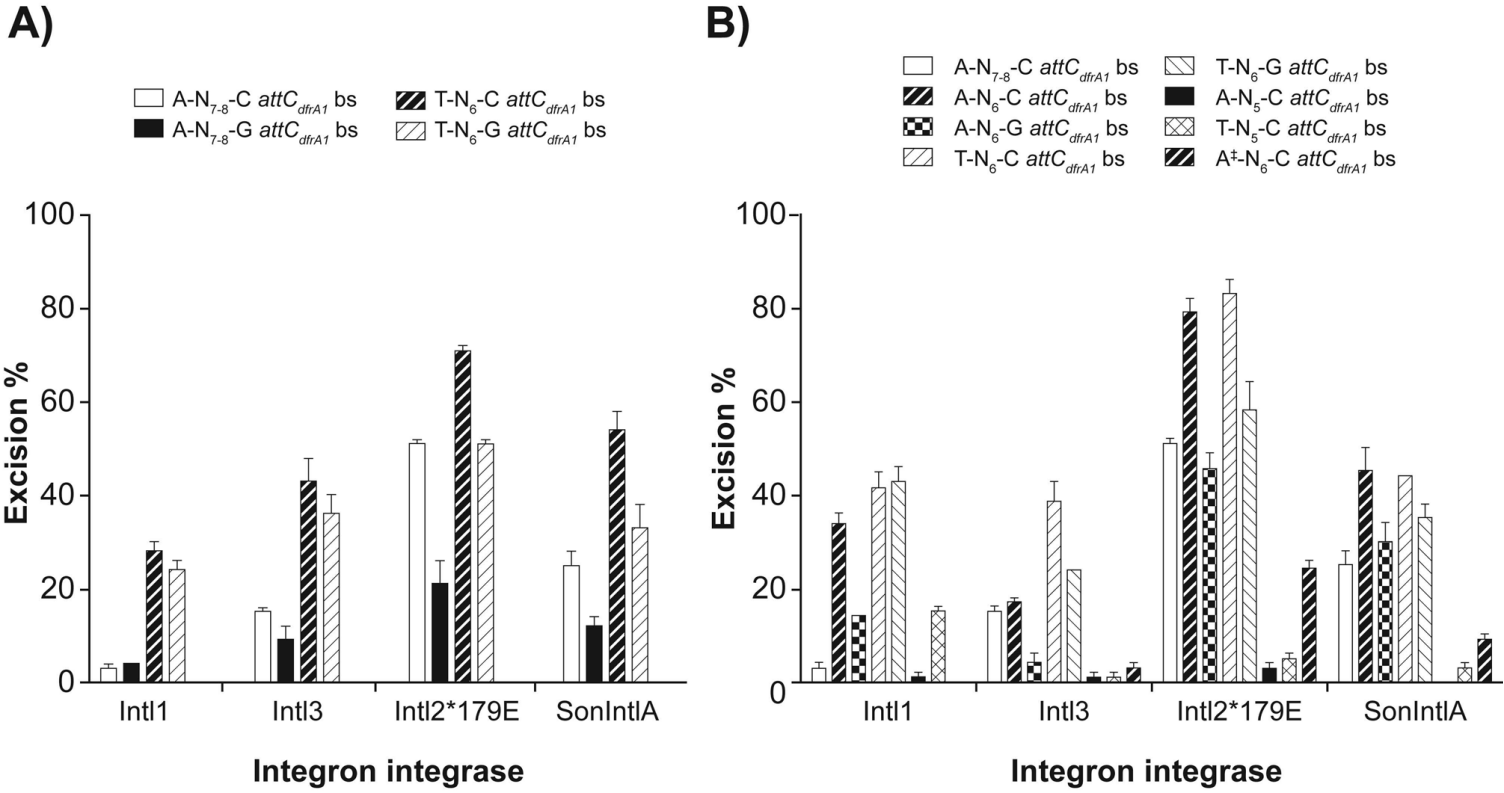
Excision percentage of *sat *cassette by the IntI1, IntI3, IntI2*179E and SonIntIA integron integrases. For each integrase, the bars indicate the excision percentage determined in the *in vivo *quantitative excision tests. Excision percentages correspond to the average of two independent assays. We tested the excision percentage of the gene cassette *sat2 *[pLQ430 and several mutants (A: first set of mutants and B: second set of mutants) created from the clone pLQ430 carrying the *attC_dfrA1 _*+ *sat2 *cassette] coding for streptothricin resistance. The ‡ indicates that the first extrahelical base is located on the opposite side of the folded bottom strand structure. Error bars show standard error.

The first set of mutants was made using various substitutions to determine whether the presence of a cytosine or a guanine at position 80 of the *attC_dfrA1 _*bottom strand (bs) could alter recognition and excision by IntI1, IntI3, IntI2*179E and SonIntIA (Figure 6A). First, we compared the ability of the four IntIs to carry out cassette excision on clones pLQ430 (A-N_7-8_-C *attC_dfrA1 _*+ *sat2*) and pAL4316 (A-N_7-8_-G *attC_dfrA1 _*+ *sat2*). The results of our QN-ETs showed that the excision activity of IntI1 remained very weak on the *sat2 *cassette when we changed the cytosine at position 80 to guanine, keeping adenines located at positions 71 and 72 of the *attC_dfrA1 _*site and the distance between the extrahelical bases at seven or eight nucleotides (Figure 7A). The efficiency of recognition and excision of this mutant cassette by IntI3 was only slightly decreased. However, the excision by IntI2*179E and SonIntIA were decreased from 51% to 21% and from 25% to 12%, respectively, by the C80G substitution of the *attC_dfrA1 _*site.

We also compared the excision activity of IntI1, IntI3, IntI2*179E, and SonIntIA on clones pAL4318 (T-N_6_-C *attC_dfrA1 _*+ *sat2*) and pAL4319 (T-N_6_-G *attC_dfrA1 _*+ *sat2*). These mutants were made by A22T substitution, and A22T and C80G substitutions, respectively, on *attC_dfrA1_*. When the first extrahelical base is a thymine (at position 73) and the distance between the extrahelical bases is six nucleotides, the C80G substitution slightly decreased excision by IntI1 and IntI3. The decrease was more pronounced with IntI2*179E and SonIntIA, from 71% to 51% and from 54% to 33%, respectively. Together, these results suggest that the presence of a cytosine as the second extrahelical base favors cassette excision by IntI, in particular by IntI2*179E and SonIntIA. These QN-ETs therefore supported our hypothesis that IntIs differ in their preferences for the extrahelical bases.

We then tested several other mutants of the *attC_dfrA1 _*site, made in order to eliminate ambiguities in the spacing of the extrahelical bases. The second set of mutants was based upon double deletions of bases A22 and T23 and A72 and T73 from the wild-type bs of the *attC_dfrA1 _*site. This forced the spacing to be six bases and made these *attC*s more comparable to T-N_6_-G *attC*s (Figure 6B). Several other substitutions were added and tested to determine whether the presence of an adenine or a thymine at position 69 or the presence of a cytosine or a guanine at position 76, of the *attC_dfrA1_*ΔAT22-ΔAT72 site (corresponding to positions 72 and 80 of the wild type site) could alter its recognition by IntI1, IntI3, IntI2*179E, and SonIntIA (Figure 6B).

The results of our QN-ETs showed that a reduction of the distance between the extrahelical bases of the *attC_dfrA1 _*site (A-N_6_-C instead of A-N_7-8_-C) led to a significant increase of excision by IntI1, IntI2*179E, and SonIntIA, while the activity of IntI3 was unchanged (Figure 7B). This preference for a shorter distance between the extrahelical bases was particularly marked for the IntI1 integrase. The excision activity of IntI1 increased from 3% to 33.5% while that of IntI2*179E increased from 51% to 79% and that of SonIntIA increased from 26% to 45% when the distance between the extrahelical bases is six nucleotides rather than seven or eight.

Mutations of the extrahelical bases to those of the consensus of the *attC*s most easily excised by IntI1 (A69T and C76G) were tested individually and in combination on the *attC_dfrA1_*∆AT22-∆AT72 site (clone pAL4305: A-N_6_-C). The A69T substitution increased excision of the *sat2 *cassette by IntI1 and IntI3, from 33.5% to 41.5% and from 17% to 38.5%, respectively, when a cytosine is present at position 76, whereas excision by IntI2*179E and SonIntIA was unchanged. The C76G substitution significantly decreased excision of this cassette by IntI1 (33.5% to 14%), IntI3 (17% to 4%), IntI2*179E (79% to 45.5%) and SonIntIA (45% to 30%) when an adenine is located at position 69. The combination of the A69T and C76G substitutions (clone pAL4311) increased excision of the *sat2 *cassette by IntI1 (33.5% to 43%) and IntI3 (17% to 24%) while it decreased excision by IntI2*179E and SonIntIA, from 79% to 58% and from 45% to 35%, respectively.

Comparison of the excision activity of the four IntIs on clones pAL4310 (A-N_6_-G *attC_dfrA1 _*+ *sat2*) and pAL4311 (T-N_6_-G *attC_dfrA1 _*+ *sat2*) showed that the efficiency of IntI1, IntI3, IntI2*179E, and SonIntIA in excision of the *sat2 *cassette was increased by the A69T substitution when the second extrahelical base is a guanine and the distance between the extrahelical bases is six nucleotides. Comparison of excision on clones pAL4308 (T-N_6_-C *attC_dfrA1 _*+ *sat2*) and pAL4311 (T-N_6_-G *attC_dfrA1 _*+ *sat2*) showed that the activity of IntI1 was unchanged by the C76G substitution when the first extrahelical base is a thymine and the distance between the extrahelical bases is six nucleotides. However, the C76G substitution significantly decreased excision by IntI3, IntI2*179E and SonIntIA, from 38.5% to 24%, 83% to 58% and 44% to 35%, respectively, when a thymine is at position 69 of the *attC_dfrA1 _*site.

The VchIntIA-VCR_bs _three-dimensional structure shows that IntIs interact closely with the extrahelical bases T12" and G20" [29]. We, therefore, tested three other mutant cassettes to determine whether variation of the distance between the extrahelical bases could alter interaction between IntIs and their substrates. The results of our QN-ETs using clones pAL4307 (A-N_5_-C *attC_dfrA1 _*+ *sat2*) and pAL4309 (T-N_5_-C *attC_dfrA1 _*+ *sat2*) showed that IntI1 can excise the *sat2 *cassette (15% excision) when the *attC_dfrA1 _*site located upstream contains the extrahelical bases T and C separated by five nucleotides (pAL4309) but not when the extrahelical bases are A and C (pAL4307) separated by the same distance. However, the IntI3, IntI2*179E and SonIntIA integrases were very inefficient in excision of this cassette with either of these clones.

Folded bottom strand *attC*s found in integrons are characterized by two extrahelical bases located on the same side of the structure. In order to determine if IntIs can recognize and excise cassettes containing an *attC *site characterized by two extrahelical bases located on either side of the folded bs structure, we changed the position of the first extrahelical base and tested the excision activity of IntI1, IntI2*179E, IntI3 and SonIntIA. Johansson *et al. *[27] previously reported that binding to the *attC_aadA1 _*bs by IntI1 was decreased by deletion of the T32 extrahelical base and by insertion of a T or an A between positions 16 and 17 to generate a bulge on the opposite side of a potential stem-loop. They showed that the presence of an adenine between positions 16 and 17 weakly affects the binding by IntI1 while the presence of a thymine significantly decreases its binding [27]. However, they did not test the excision activity of IntI1 on a cassette containing this mutant *attC *site. Our QN-ETs using clone pAL4304 (A^‡^-N_6_-C *attC_dfrA1 _*+ *sat2*: the ‡ indicates that the first extrahelical base is located on the opposite side of the folded bottom strand structure; the two extrahelical bases are located on either side of the folded bs structure) showed that IntI1 was not able to excise the *sat2 *cassette when the altered A^‡^-N_6_-C *attC_dfrA1 _*site is located upstream, while IntI3 showed an excision activity of only 3%. However, the IntI2*179E integrase showed an excision activity of 24% on this very unusual *attC *site, while SonIntIA showed a level of excision of 9%.

## Discussion

The dissemination of antibiotic resistance by mobilization of resistance gene cassettes is a key factor affecting the clinical usefulness of antibiotics. The recruitment of these cassettes by integrons is carried out by IntIs and the efficiency of recombination by these enzymes varies greatly. In this work, we studied some parameters that affect the specificity of these recombinases for *attC *sites.

### Influence of extrahelical base identity, spacing and position on specificity of IntIs

#### Extrahelical base identity

The data presented in this study showed that the IntI1 was efficient in cassette excision using T-N_6_-G or T-N_6_-C *attC *sites, while IntI3 recognized a limited range of *attC*s and recombined mainly cassettes with T-N_6_-C *attC *sites (Figure 7). For their part, IntI2*179E and SonIntIA tolerated changes to the identity of extrahelical bases, as they efficiently excised cassettes with *attC*s characterized by most of the extrahelical base combinations tested (A-N_6_-C, A-N_6_-G, T-N_6_-C and T-N_6_-G). In their IntI binding study, Johansson and colleagues [27] showed that substitution of the first extrahelical base (T) with a cytosine or an adenine, and substitutions of the second extrahelical base (G) with any of the alternative bases, does not affect binding of IntI1 to the *attC_ant(3")-Ia _*site. However, they did not test the excision efficiency of IntI1 on these mutant cassettes. Taken together, these results suggest that the extrahelical base identities do not affect the binding of IntIs but do influence the recombination reaction. They confirm our hypothesis that *attC *preferences of IntIs are related to the recognition of the extrahelical bases.

Surprisingly, our results showed that the presence of a cytosine rather than a guanine at the second extrahelical base position increased cassette excision by IntI3, IntI2*179E and SonIntIA, whether the first extrahelical base is a thymine or an adenine. The excision activity of IntI1 was increased by the presence of a cytosine at the second extrahelical position only when the first extrahelical base is an adenine. These results were unexpected since most *attC *sites have a guanine at the second extrahelical position. They suggest that the emergence of cassettes with increased mobility is possible. The only example of a cassette containing an *attC *site with a cytosine as the second extrahelical base is *bla_VIM-2_*, but its mobility remains to be evaluated. Results recently obtained by Bouvier *et al. *[34] show that the *attC *x *attC *recombination (*attC_aadA7 _*x VCR) carried out by IntI1 is slightly increased by the guanine (G) to cytosine (C) substitution of the second extrahelical base of the VCR when the first extrahelical base is a thymine. In their study, mutations were made on the downstream *attC *partner whereas they were made on the upstream *attC *partner in this study. Our QL-ETs and results obtained by Bouvier *et al. *[34] suggest that changes to the downstream *attC *partner may have a greater impact on the ability of IntIs to excise cassettes.

#### Distance between the extrahelical bases

Since the VchIntIA-VCR_bs _three-dimensional structure shows that IntIs interact closely with the extrahelical bases T12" and G20" [29], we tested the effect of the distance between these bases on this interaction. Our data showed that IntI1, IntI3, IntI2*179E and SonIntIA most efficiently excised cassettes containing *attC*s when the spacing between the extrahelical bases was six nucleotides. They also showed that IntI2*179E and SonIntIA were more tolerant than IntI1 and IntI3 to changes in spacing between the extrahelical bases. Johansson *et al. *[27] showed that increasing the distance between the two extrahelical bases (from six to eight or 10) does not affect binding of IntI1 to the *attC_ant(3")-Ia _*site, but they did not test the recombination activity of IntI1 on these mutant *attC *sites. It appears that the distance between the extrahelical bases is important for the excision reaction but not for bs *attC *binding by IntIs.

#### Position of the first extrahelical base

As mentioned above, Johansson *et al. *[27] reported that the presence of an adenine between positions 16 and 17 on the opposite side of a potential stem-loop combined with the deletion of the T32 extrahelical base decreased binding to the *attC_aadA1 _*bs by IntI1. In our study, we observed that, despite the fact that their excision activity is decreased, IntI2*179E and SonIntIA can excise the *sat2 *cassette when the altered A^‡^-N_6_-C *attC_dfrA1 _*site, characterized by an extrahelical base at position 22 and another at position 78, is located upstream. IntI1 and IntI3 have no apparent activity on this very atypical site. The influence of the first extrahelical base position (corresponding to T32) was also tested by Bouvier *et al. *[34] and they showed that the re-localization of this base at the corresponding location on the opposite strand leads to a decrease of VCR_bs _excision by IntI1. Together, these results show that, in addition to being more tolerant to changes in the identity and spacing between the extrahelical bases, IntI2*179E and SonIntIA are more tolerant than IntI1 and IntI3 with respect to the position of the first extrahelical base. They also suggest that changing the first extrahelical base to a thymine decreases the binding by IntIs and probably affects the excision activity. However, the re-localization of the first extrahelical base as an adenine does not affect the binding but the excision activity is decreased.

### QN-ETs using the *attC_dfrA1 _*site: IntI1 versus IntI2*179E

The results of our QN-ETs using IntI1 and IntI2*179E with the T-N_6_-G *attC_dfrA1 _*site raised an important issue. It is not clear why the excision percentage observed with IntI1 on cassettes containing the T-N_6_-G *attC_dfrA1 _*site is not higher than that observed with IntI2*179E on the same substrates. Our QL-ETs showed that IntI1 is generally more effective than IntI2*179E in excision of cassettes containing T-N_6_-G *attC *sites (for example, *aac(6')-Ia*-orfG and *ant(3'')-Ia*). It is possible that these differences are explained by the presence of different nucleotides near the extrahelical bases of the *attC_dfrA1 _*mutants used for our QN-ETs and those of *attC *sites used in our QL-ETs. It has been shown that the identity of the bases located near the extrahelical bases influences the binding of IntI1 [27].

### Structural elements of IntI1, IntI2*179E, IntI3, SonIntIA and VchIntIA involved in *attC *recognition

IntIs bind specifically to the *attC *bs [26] and the extrahelical bases resulting from its folding are important for recognition by these enzymes [27,29]. The three-dimensional structure of the VchIntIA-VCR_bs _complex reveals that the extrahelical base T12'' is stabilized by *cis *interactions with the β-4,5 strands from the non-attacking subunits by becoming inserted between two stacked histidines (H240 and H241 in VchIntIA (Figure 4A); H250 and H251 in IntI1) and a highly conserved proline [P232 in VchIntIA (Figure 4A); P242 in IntI1] [29]. The attacking subunits make important DNA contacts in *trans *with the extrahelical base G20'' through interactions with Q145, W157, K209, Y210 and W219 in VchIntIA (Figure 4B) that correspond to K156, R168, K219, Y220 and W229 in IntI1 [29]. The protein-DNA interactions are otherwise essentially nonspecific [29].

**Figure 8:**
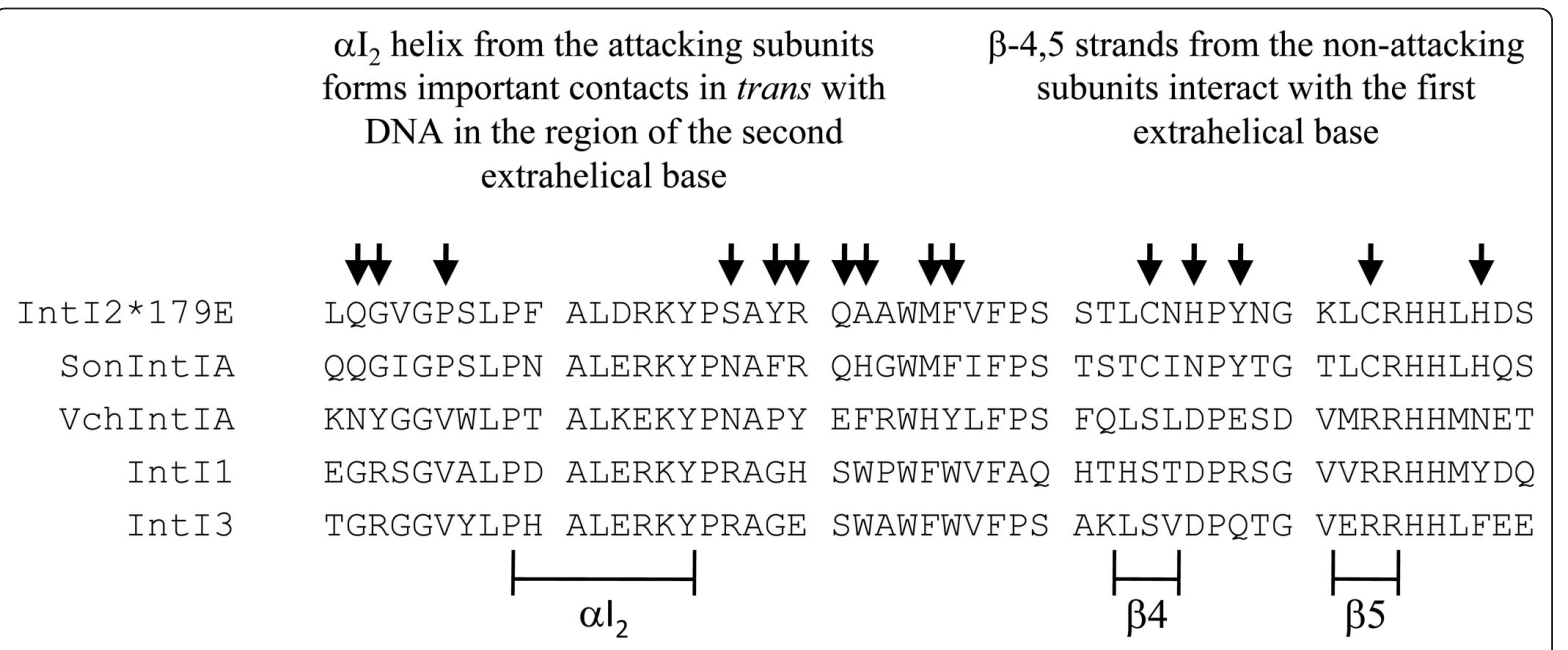
**Sequence alignment of the αI**_**2 **_**helix and the β-4,5 strands region of some integron integrases (IntIs)**. IntI2*, class 2 IntI from Tn*7*; SonIntIA, IntI from *Shewanella oneidensis *MR1; VchIntIA, IntI from the *Vibrio cholerae *chromosomal integron; IntI1, class 1 IntI from plasmid pVS1; IntI3, class 3 IntI from a *S. marcescens *plasmid. Residues potentially related to the difference in specificity of IntI2 and SonIntIA versus IntI1 and IntI3 are identified by arrows. Positions of the αI_2 _helix and the β-4,5 strands in this figure are based on the VchIntIA structure (Protein Data Bank accession no. 2A3V) [29].

We compared the region located between the αI_2 _helix and the β-4,5 strands of IntI1, IntI3, IntI2*179E, SonIntIA and VchIntIA and observed that many residues are conserved among these enzymes (Figure 8). This reflects the importance of this region in *attC *recognition by IntIs. However, we identified some differences between IntI2*179E and SonIntIA versus IntI1 and IntI3 sequences that could be responsible for the greater versatility of IntI2*179E and SonIntIA in excision of cassettes containing non-T-N_6_-G *attC*s. One interesting difference is the presence of two cysteine residues in the β-4 and β-5 strands of IntI2*179E and SonIntIA. The same positions are occupied by a serine and an arginine in IntI1 and IntI3. We previously found that the cysteine residue in the β-5 strand is essential to the excision activity of *Shewanella*-type integrases while the cysteine in the β-4 strand is less important [33]. Mutagenesis of the two cysteines studied in SonIntIA suggests that there is no disulfide bridge between the β-4 and β-5 strands of these integrases [33]. However, we do not know if these cysteine residues play a role in the ability of IntI2*179E and SonIntIA to tolerate changes to the extrahelical base identity and spacing. Other differences (indicated by arrows in Figure 8) are located at various positions between the αI_2 _helix and the β-4,5 strands of IntIs and could also contribute to the greater versatility of IntI2*179E and SonIntIA in excision of cassettes.

Some residues and motifs located outside the αI_2 _helix and the β-4,5 strands could also be related to the different preferences of IntIs for extrahelical base identity and spacing. For example, Demarre *et al. *[35] found mutations of IntI1, with higher activity on wild type and mutant *attC *sites, in the loop located between the β-1 and β-2 strands. Interestingly, the β-2 strand contains one of the two residues, R168 (W157 in the sequence of VchIntIA), that interact with the extrahelical base G20" [29]. Also, Johansson *et al. *[30] showed that substitution of the tryptophan residue at position 199 of IntI1 with alanine, which is aliphatic, small, and uncharged, decreases DNA binding. Interestingly, when it was replaced by an aromatic residue (W199Y), it regained its affinity for *attC_bs _*[30]. These results suggest that the presence of an aromatic or a bulky amino acid residue at this position is important. The authors propose that the decreased binding of the IntI1W199A mutant could be explained by structural changes of the αI_2 _helix and the β-4,5 strands region [30]. Thus, in addition to residues that interact directly with the extrahelical bases, we must also consider the residues that are important to protein structure in our attempt to identify the factors that may affect the ability of IntIs to excise cassettes. The alignment of IntIs used in our study show that the position 199 is occupied by a tryptophan residue in IntI1 and IntI3 while it is occupied by a glutamine in IntI2*179E and SonIntIA. This difference may contribute to explain the different ability to excise cassettes of these IntIs.

In summary, it seems that IntI2 and SonIntIA have an evolutionary path that is different from IntI1 and IntI3, in their ability to recognize and excise cassettes. IntI2*179E and SonIntIA, although generally less efficient in cassette excision, tolerate a wider variety of configurations of the extrahelical bases of *attC *sites. We believe that the ability of IntI2*179E and SonIntIA to excise cassettes containing *attC*s characterized by a broader range of extrahelical base identity and spacing could be related to a greater flexibility of their αI_2 _helix and their β-4,5 strand domains.

### Acquisition of new cassettes

Analysis of the variable region of class 1 integrons showed that these multiresistance integrons contain a large number of different cassettes and those containing T-N_6_-G *attC*s are found at various positions within the variable region. This can be explained by our finding that these cassettes can be easily recognized and excised by IntI1. The IntI1 integrase is efficient in cassette integration and, since cassettes are preferentially integrated by *attI *x *attC *recombination [3], cassette order tends to reflect the order of introduction of antibiotics, with the most recently acquired cassettes closest to the promoter.

Class 2 integrons, carrying the IntI2* or IntI2 integrases, as well as class 3 integrons, carrying the IntI3 integrase, have only a limited range of cassettes. Among the arrangements of cassettes associated with the class 2 integrons are *dfrA1*-*sat2*-*aadA1*-*orfX*, *estX*-*sat2*-*aadA1*-*orfX *and *sat2*-*aadA1*-*orfX *[18]. The position of cassettes within this class of multiresistance integrons is conserved since most class 2 integrons carry the inactive IntI2* integrase. Recently, class 2 integrons with active integrases have been found [36,37] but they still have a relatively limited number of cassette arrangements. The *dfrA1 *and *estX *cassettes contain non-T-N_6_-G *attC*s while the *sat2*, *aadA1 *and *orfX *cassettes contain T-N_6_-G *attC*s. Two different arrangements of resistance gene cassettes were found to be associated with class 3 integrons: *bla_IMP-1_*-*aac(6')-Ib *and *bla_GES-1_*-*bla_OXA_*/*aac(6')-Ib *[38,39]. The *bla_GES_*_-1 _cassette contains a T-N_6_-G *attC *site while *bla_IMP-1_*, *aac(6')-Ib*, and *bla_OXA_*/*aac(6')-Ib *cassettes contain non-T-N_6_-G *attC*s.

The cases of the *dfrA1*, *bla_IMP-1 _*and *bla_GES-1 _*cassettes are particularly interesting. Although first found in class 2 and class 3 integrons, these cassettes are now more frequently disseminated by class 1 integrons. This may reflect the greater versatility of IntI1 in cassette rearrangement. Moreover, the *dfrA1 *cassette is nearly always located in first position in class 1 integrons. As we have shown, cassettes containing non-T-N_6_-G *attC*s are weakly excised by IntI1, which suggests that integration of cassettes containing *attC *sites like that of *dfrA1 *may hinder their own subsequent excision and that of their downstream neighbors (*attC *x *attC *excision). In the case of the *bla_IMP-1 _*cassette, the additional secondary structure located between the extrahelical bases of the *attC *bs does not interfere with the ability of IntIs to excise cassettes when this recombination site is located either upstream or downstream of the gene (Table 1). The *bla_GES-1 _*cassette is associated with a T-N_6_-G *attC *site that would facilitate its acquisition by integrons (in particular, class 1 integrons) and its dissemination among bacteria.

Leon and Roy [40] have shown that there is no relationship between a cassette structural gene and its associated *attC *site. According to their new model for gene cassette formation, group IIC-*attC *introns can target separately a transcriptional terminator adjoining a gene and an isolated *attC*. Thereafter, the gene and the *attC *can be joined by homologous recombination between the introns, followed by transcription, RNA splicing, and reverse transcription to lead to the formation of a cassette [40]. The characteristics of the structure of the bottom strand of the *attC *site would determine the subsequent mobility of the cassette.

## Conclusions

In conclusion, this work and previous studies [27,29,32,34] clearly show that the *attC *structure is an important factor that facilitates the integration of new cassettes into integrons. In our study, we carried out excision tests with several IntIs on cassettes containing a wide variety of *attC *sites. This work could aid the development of a site-specific recombination system using the IntIs. In contrast to the Cre recombinase of the Cre-*lox *system, IntIs have a more relaxed specificity for their recognition sites *attI *and *attC*. The main advantage of a site-specific recombination system using an IntI is that it would allow insertion of genes (cassettes) in tandem. The results presented in this article could be used to optimize such a system.

## Methods

### Bacterial strains and growth media

*Escherichia coli *strains were cultured at 37°C in Luria-Bertani (LB) broth or on LB agar supplemented with ampicillin (100 μg/mL; Sigma, MO, USA), chloramphenicol (50 μg/mL; Sigma) or streptothricin (3 μg/mL). DH5α cells [F^- ^*endA1 glnV44 thi-1 recA1 relA1 gyrA96 deoR nupG*Φ80d*lacZ∆*M15 ∆(*lacZYA-argF*)U169 *hsdR17*(*r_K_*^- ^*m_K_^+^*) λ^-^] were used as a host for construction and maintenance of all plasmid clones and for QL-ETs, while HB101 cells [F^- ^*mcrB mrr hsdS20*(r_B_^- ^m_B_^-^) *recA13 leuB6 ara-14 proA2 lacY1 galK2 xyl-5 mtl-1 rpsL20*(Sm^R^) *glnV44 λ*^-^] were used for QN-ETs.

### Bioinformatic analysis

Sequence analysis was done using the Genetics Computer Group programs (Wisconsin Package version 10.3; Accelrys). Folding of *attC *bottom strands was done using the MFOLD software (http://mfold.bioinfo.rpi.edu/cgi-bin/dna-form1.cgi).

### Mutagenesis method

Several mutations were introduced within the *attC_dfrA1 _*site located upstream of the *sat2 *cassette cloned into pACYC184 (clone pLQ430). Specific mutations were introduced into pLQ430 using the QuickChange site-directed mutagenesis system including *Pfu *Turbo (Stratagene, CA, USA) DNA polymerase. Primer pairs, designed with the OLIGO software package (version 4.1; National Biosciences, MN, USA), were used to create 16 mutants of the *attC_dfrA1 _*site. The forward primers are shown in Additional File 1. Mutagenesis products were digested with DpnI, transformed into *E. coli *DH5α, grown in LB medium for 1h and selected for chloramphenicol resistance by plating on LB agar plates containing chloramphenicol. DNAs from several colonies were purified using a QIAprep spin miniprep kit (Qiagen, Düsseldorf, Germany) and sequenced to confirm the presence of desired mutations and the integrity of surrounding sequences. The isolateswere maintained as glycerol stock cultures at -80°C.

### Qualitative excision tests (QL-ETs)

IntI1, IntI3, IntI2*179E, SonIntIA and VchIntIA clones (see Additional File 2) were introduced by transformation into *E. coli *DH5α containing various cassettes cloned into pACYC184 (Table 1). *E. coli *was grown in LB medium at 37°C to an optical density at 600 nm of 0.5. Cassette excision was induced by the overexpression of the integrase gene using 1 mM isopropyl-β-D-thiogalactopyranoside (IPTG; Sigma) and incubation at 37°C overnight. Cell cultures were done in the presence of ampicillin and chloramphenicol. Plasmid DNA was subsequently extracted from 5-ml cultures with a QIAprep spin miniprep kit (Qiagen).

In order to determine the ability of IntIs to excise cassettes, we used polymerase chain reaction (PCR) primers pACYC184-5' and pACYC184-3' (See Additional File 1) to detect reductions in length of cassette clones. PCR conditions were 5 min at 95°C, 30 cycles consisting of 30 s at 95°C, 30 s at 62°C and 3 min 30 s at 68°C, and a final elongation step of 5 min at 68°C.

### Quantitative excision tests (QN-ETs)

Cells containing integrase clones were transformed by various plasmids containing gene cassettes cloned into pACYC184 (See Additional File 2). One colony of each double transformant was used to inoculate 5 mL of LB medium and grown at 37°C to an optical density at 600 nm of 0.5. Cell cultures were done in the presence of ampicillin and chloramphenicol. Isopropyl-β-thio-galactoside (IPTG) was then added to a final concentration of 1 mM to induce cassette excision and cultures were incubated at 37°C overnight; plasmid DNA extractions (Qiagen) were done on each culture. DNA was incubated at 37°C 1h with PstI to digest the integrase clone and prevent its co-transformation into the *E. coli *strain used to determine, by replica plating, the antibiotic resistance cassettes that were excised. Thereafter, cassette clones were transformed into *E. coli *HB101 and colonies selected for chloramphenicol resistance.

One hundred colonies of each transformation were replicated on LB chloramphenicol + streptothricin plates and incubated at 37°C overnight. The proportion of transformants that could not grow indicated the excision percentage of the *sat2 *cassette for each integrase and each upstream *attC *tested.

## Abbreviations

A: adenine; Arg: arginine; bs: bottom strand; C: cytosine; G: guanine; His: histidine; IntI: integron integrase; LB: Luria-Bertani; Lys: lysine; N: nucleotide; QL-ETs: qualitative excision tests; QN-ETs: quantitative ETs; R: arginine (amino acid symbol) or purine (nucleotide symbol); T: thymine; Tn: transposon; Trp: tryptophan; Tyr: tyrosine; VCR: *Vibrio cholerae *repetitive DNA sequence; W: tryptophan; Y: pyrimidine.

## Competing interests

The authors declare that they have no competing interests.

## Authors' contributions

AL performed the experiments and wrote the article. PHR supervised the work and participated in writing the article.

## Supplementary Material

Additional File 1 **Primers used in this study**. Click here for file

Additional File 2 **Integrase clones and mutant cassette clones used in this study**. Click here for file
