# Supplementary material for: Effect of attC structure on cassette excision by integron integrases
Source: Mob DNA. 2011 Feb 18;2:3. doi: 10.1186/1759-8753-2-3 (PMC3053210; doi:10.1186/1759-8753-2-3)
Supplement: Additional File 1 — Primers used in this study. [file 1759-8753-2-3-S1.DOC]

## Additional file 1: Primers used in this study.

| **Primer** | **Nucleotide sequence (5′ to 3′)** | **Annealing temperature (ºC)** |
| --- | --- | --- |
| pACYC184-5′ | TGTAGCACCTGAAGTCAGCC | 62 |
| pACYC184-3′ | ATACCCACGCCGAAACAAG | 62 |
| *attC_dfrA1_*C80G | GGTTAACAAGTGGCACCAACGGATTCGCAAAC | 58 |
| *attC_dfrA1_*A72T | GCAGCAACGGAATCGCAAACCTG | 54 |
| *attC_dfrA1_*A71T-C80G | GGTTAACAAGTGGCACCAACGGAATCGCAAAC | 60 |
| *attC_dfrA1_* ΔAT72 | GTGGCAGCAACGGTCGCAAACCTGTC | 56 |
| *attC_dfrA1_* ΔAT22 | GCCAGGTTTGCGCCGCTGTGCCAG | 54 |
| *attC_dfrA1_*C16G | CAGGTTTGCGATCCGCTCTGCCAGGCGTTAG | 64 |
| *attC_dfrA1_*ΔAT72-GC79CG | GGGTTAACAAGTGGCACGAACGGTCGCAAAC | 62 |
| *attC_dfrA1_*A71T-ΔAT72 | GTGGCAGCAACGGACGCAAACCTGTCAC | 62 |
| *attC_dfrA1_*A71T-ΔAT72-GC79CG | GTGGCACGAACGGACGCAAACCTGTCA | 62 |
| *attC_dfrA1_*C16G-ΔAT22 | CAGGTTTGCGCCGCTCTGCCAGGC | 62 |
| *attC_dfrA1_*GC79CG | CAAGTGGCACGAACGGATTCGCAAAC | 62 |
| *attC_dfrA1_*A22T | GCCAGGTTTGCGAACCGCTGTGCC | 54 |
| *attC_dfrA1_*C16G-A22T-GC79CG | GCCAGGTTTGCGAACCGCTCTGCC | 54 |
