# Supplementary material for: Effect of attC structure on cassette excision by integron integrases
Source: Mob DNA. 2011 Feb 18;2:3. doi: 10.1186/1759-8753-2-3 (PMC3053210; doi:10.1186/1759-8753-2-3)
Supplement: Additional File 2 — Integrase clones and mutant cassette clones used in this study. [file 1759-8753-2-3-S2.DOC]

Additional file 2: Integrase clones and mutant cassette clones used in this study.

| **Plasmid** | **Description** | **Reference** |
| --- | --- | --- |
| pLQ419 | *intI1* cloned into pTrc99a | (F. Gagnon and P. H. Roy, unpublished) |
| pLQ421 | *intI2***179E* cloned into pTrc99a | [17] |
| pLQ422 | *intI3* cloned into pTrc99a | (F. Gagnon and P. H. Roy, unpublished) |
| pFD03 | Son*intIA* cloned into pTrc99a | [16] |
| pLQ661 | Vch*intIA* cloned into pTrc99a | (N. Messier and P. H. Roy, unpublished) |
| pAL4301 | *attC_dfrA1_*C80G bs + *sat2* cloned into pACYC184 | This study |
| pAL4302 | *attC_dfrA1_*A71T bs + *sat2* cloned into pACYC184 | This study |
| pAL4303 | *attC_dfrA1_*A71T-C80G bs + *sat2* cloned into pACYC184 | This study |
| pAL4304 | *attC_dfrA1_*ΔAT72 bs + *sat2* cloned into pACYC184 | This study |
| pAL4305 | *attC_dfrA1_*ΔAT22-ΔAT72 bs + *sat2* cloned into pACYC184 | This study |
| pAL4306 | *attC_dfrA1_*ΔAT22-ΔAT72-C80G bs + *sat2* cloned into pACYC184 | This study |
| pAL4307 | *attC_dfrA1_*ΔAT22-ΔAT72-GC79CG bs + *sat2* cloned into pACYC184 | This study |
| pAL4308 | *attC_dfrA1_*ΔAT22-ΔAT72-A71T bs + *sat2* cloned into pACYC184 | This study |
| pAL4309 | *attC_dfrA1_*ΔAT22-ΔAT72-A71T-GC79CG bs + *sat2* cloned into pACYC184 | This study |
| pAL4310 | *attC_dfrA1_*ΔAT22-ΔAT72-C16G-GC79CG bs + *sat2* cloned into pACYC184 | This study |
| pAL4311 | *attC_dfrA1_*ΔAT22-ΔAT72-C16G-A72T-GC79CG bs + *sat2* cloned into pACYC184 | This study |
| pAL4313 | *attC_dfrA1_*ΔAT22-ΔAT72-G18A bs + *sat2* cloned into pACYC184 | This study |
| pAL4316 | *attC_dfrA1_*C16G-GC79CG bs + *sat2* cloned into pACYC184 | This study |
|  |  |  |
| pAL4317 | *attC_dfrA1_*C16G-A71T-GC79CG bs + *sat2* cloned into pACYC184 | This study |
| pAL4318 | *attC_dfrA1_*A22T bs + *sat2* cloned into pACYC184 | This study |
| pAL4319 | *attC_dfrA1_*C16G-A22T-GC79CG bs + *sat2* cloned into pACYC184 | This study |
